# International data-sharing norms: from the OECD to the General Data Protection Regulation (GDPR)

**DOI:** 10.1007/s00439-018-1919-7

**Published:** 2018-08-01

**Authors:** Mark Phillips

**Affiliations:** Centre of Genomics and Policy, McGill University, Montreal, QC H3A 0G1 Canada

## Abstract

The evolution of genomic research and its integration into clinical practice, as they become international—even global—endeavors, has brought us to a place where scientists and clinicians may now only ignore the rules governing international data sharing at their own peril. Open data policies, on the one hand, increasingly require custodians of others’ genomic data to make it as widely available as feasible, including to researchers in other countries. Data protection law, on the other, has become a significant hurdle to the sharing of personal data across jurisdictional borders. The space between these two competing duties is narrowing. In contrast with the other texts in this volume, which explore the present and future of data sharing and data protection, this article’s focus is on the past. It centres on the historical development of the data protection rules regarding the international transfer of personal data up to the present. The article’s aim is to bring into focus the underlying objectives that have influenced and that will continue to influence the way that data protection rules are applied to the fields of genomics and health, as well as future developments in data protection generally. The first part of this article describes the development of international data-sharing data protection rules since 1970. The second considers difficulties in applying general data protection rules to the specific context of genomics and health. The third and final part compares the options available to comply with the international transfer restrictions set out in the standard-setting EU General Data Protection Regulation from a genomics perspective.

## From the OECD to the GDPR

International data-sharing norms evolved in tandem with the broader field of personal data protection within which it forms a well-defined component. Since its inception, data protection has, in turn, been driven by the development of information technology.

The law considered to be the first data protection statute, adopted in 1970 in the German federal state of Hessen, did not address international data transfers (Kuner 2013). But those that followed, both within Europe and elsewhere, soon recognized that it was pointless to establish a framework to protect personal data if those protections could be effectively circumvented by simply moving the data of the people it was designed to protect to another jurisdiction. But this recognition immediately came into tension with an existing tendency against restrictions on international data transfer, given the noxious impact of these restrictions on communications, commerce, science, and any number of other human endeavors (Ploman 1982). As the following paragraphs describe, the balance was found by aiming to harmonize meaningful data protection across jurisdictions to enable the removal of transfer restrictions, which would then become redundant.

This perspective is implicit in two of the first data protection frameworks to have international ambitions and to achieve global influence, namely the Organization for Economic Co-operation and Development Privacy Guidelines in 1980 (OECD 2013) and the Council of Europe Convention for the protection of individuals with regard to automatic processing of personal data (Council of Europe 1981), known as Convention 108.

Since the drafters of these two early initiatives coordinated their efforts (Dove and Phillips 2015), it is unsurprising that they each arrived at similar positions. Both instruments’ treatment of international transfers is framed around the idea that such transfers should generally be presumed to be allowed, with Convention 108 going so far as to prohibit restrictions on transfer solely for reasons of privacy between participating states, except in the case of sensitive data, which must even then remain prohibited if the receiving jurisdiction provides “equivalent protection” [Council of Europe 1981: Article 12(3)(a)].

Both instruments do also, however, recognize transfer restrictions as being valid when the protections they provide risk being circumvented by the lack of protection in force in the jurisdiction of the recipient or an intermediary entity (Council of Europe 1981: Article 12, OECD 2013: Article 17).

Despite the similar perspective, a distinction can be discerned between the two frameworks’ approaches to transfer that would ultimately develop into the division between the leading adequacy and accountability approaches to regulating international transfer of personal data. While Convention 108 implicitly established a definitive list of jurisdictions to which personal data could generally be transferred (i.e., other participating states), the OECD Privacy Guidelines allowed restrictions to be imposed with respect to transfers to a country that “does not yet substantially observe these Guidelines” (OECD 2013: Article 17), a determination open to much more interpretation from the perspective of an organization attempting to determine whether a desired transfer is permitted.

In broad strokes, adequacy requires transfers to conform to a mechanism that has been approved in advance by an authority to declare that it guarantees protection at an acceptable level in the foreign jurisdiction. Accountability, on the other hand, requires the entity wishing to transfer personal data to make its own ad hoc assessment and to determine for itself the protections necessary for the transfer to be permissible.

The adequacy approach was cemented by later developments in Europe, and in particular by the European Union’s Data Protection Directive (1995) and its successor, the General Data Protection Regulation (European Union 2016). This approach requires that any transfer to a country outside the European Union must be made in accordance with a transfer justification that has been approved in advance by the European Commission. The preferred mechanism is prior Commission approval of a foreign legal framework. These approvals are referred to as adequacy decisions. When personal data will remain subject to a framework that has been deemed adequate, transfer requires no further justification. The reliance on adequacy decisions and alternative transfer justifications permitted within the EU frameworks are discussed in the context of health data sharing below in part III of this article.

Data protection statutes that are influenced by the OECD Privacy Guidelines often take the accountability approach. Canada’s Personal Information Protection and Electronic Documents Act (PIPEDA), for example, is closely modeled on the principles set out in the Privacy Guidelines, and provides that an organization subject to it “is responsible for personal information in its possession or custody, including information that has been transferred to a third party for processing” (Canada 2000: Schedule 1, Principle 4.1.3). An inadequate foreign data protection regime does not necessarily preclude transfer, as it is possible to “use contractual or other means to provide a comparable level of protection while the information is being processed by a third party” (Canada 2000: Schedule 1, Principle 4.1.3).

The Asia-Pacific Economic Cooperation (APEC) forum’s 2005 APEC Privacy Framework is a business-oriented data protection framework heavily inspired by the OECD Guidelines. Its Article IX accordingly adopts the accountability principle for data transfer. APEC has demonstrated a sustained commitment to ensuring the relevance of the Privacy Framework despite that it initially attracted limited engagement even among APEC’s 21 members throughout the Pacific Rim, which include Australia, Hong Kong, and the three North American economies (Heyder 2014).

In 2011, Cross-Border Privacy Rules (CBPRs) were announced as a mechanism to bring increased certainty to the APEC Privacy Framework’s transfer rules (APEC 2011). Instead of requiring senders to determine, based on the entirety of the circumstances, whether appropriate protection will be maintained with the data in the recipient’s control, CBPRs are codes of conduct that businesses can be certified as conforming to, to demonstrate that they have implemented protections consistent with the APEC Privacy Framework for the purpose of acting as personal data transfer recipients. This arguably moves the model closer to an adequacy model, although its closer relative is likely the U.S./EU Privacy Shield (and its predecessor, the Safe Harbour), in that each is effectively a self-certification regime driven by the business sector. Although CBPR certification includes a measure of independent review prior to approval by what it refers to as an Accountability Agent, this evaluation centres on checking the applicant’s responses to a questionnaire against CBPR program requirements.

Despite the fact that both the APEC Privacy Framework and its CBPR system are voluntary regimes with no independent legal effect, the CBPR regime does rely on the possibility of recourse to enforcement bodies against businesses who have indicated they are in compliance. In 2016, for example, the United States Federal Trade Commission announced its first CBPR enforcement action after it charged a business with falsely claiming to been CBPR certified (Federal Trade Commission 2016).

Another data protection legal framework that has international influence in practice, and that is specific to health data, is the one incorporated in the United States’ Health Insurance Portability and Accountability Act of 1996 (HIPAA). A number of information technology service providers that may hold data in the cloud outside the United States, or that are based outside of the United States, for example, advertise themselves as being HIPAA compliant (Maheu 2014).

HIPAA establishes that when the entities that are subject to it, which it refers to as “covered entities”, enlist the services of third parties to carry out their healthcare activities and functions, these third parties generally must meet the data protection requirements that HIPAA sets out for “business associates” (United States 1996, p 160, 164), which is the name it gives to these third parties.

These rules apply irrespective of whether health data crosses international boundaries through the process. If anything, HIPAA’s approach to transfer is notable for its apparent indifference to the jurisdiction in which the personal health data finds itself. Business associate arrangements centre on whether binding safeguards have been provided for through contractual means, but HIPAA does also provide for direct liability of business associates in some circumstances. HIPAA, however, differs from some other US laws in that it has no explicit extraterritorial application, and so its practical protection of personal health data held abroad may be unclear.

Health-sector specific data protection frameworks are also beginning to emerge on the international scale. In the international private sector, for example, the World Anti-Doping Agency’s International Standard for the Protection of Privacy and Personal Information (2018) is an ambitious Code aimed at balancing Anti-Doping Agencies’ public transparency and accountability with the protection of privacy. Interestingly, this international Code pays scarcely any attention to the issue of international transfer of personal data at all, perhaps based on the rationale that guaranteeing uniform personal data protection around the globe, as the Code seems to aspire to do, in turn makes the regulation of transfer moot.

Beyond health-specific or regional data protection frameworks, initiatives toward a truly general and global regime remain limited. Despite scattered efforts, such as the 2009 Madrid Resolution and more recent arguments that the United Nations should take the lead in this respect, little progress has been made, and if anything the GDPR has emerged as the closest contender to a globally recognized framework (Public Voice 2009; de Hert and Papakonstantinou 2016).

The underlying goal of establishing consistent data protection across the globe, therefore, remains aspirational. Because this goal has historically been seen as the precondition for eliminating restrictions on international transfer, regulation of transfer will persist for the foreseeable future. The questions now are first, whether special considerations apply in the context of health and genomic data, and second, how translational health and genomics projects should meet their transfer obligations, especially those imposed by the GDPR.

## Genomics through a data protection lens

The uncertainty in data protection in general, and with respect to international transfer in particular, can be compounded when data protection rules formulated to be as general as possible, as sketched in the previous section of this article, are applied to the quite specific context of genomic data transfer in particular.

There is some debate, however, about whether rules specific to the genomic context are necessary. One side of this debate has argued that this approach reflects an overly emotional response to the issue. This current evokes an earlier debate around “HIV exceptionalism”, a phrase coined to refer to the idea that, given the particular stigma attached to HIV and AIDS, that specific protections should be provided to patients, particularly around confidentiality, while detractors argue that these protections do more harm than good (Oppenheimer and Bayer 2009). Attempts have since been made by genetic researchers and their advocates to establish a notion of “genetic exceptionalism”, to make the somewhat analogous case that specific regulation targeting genetic data is distorted by ill-informed public squeamishness, and to focus instead on ensuring protection through existing general rules in data protection and other areas (Evans and Burke 2008). This current has generally been outspoken against genetic nondiscrimination legislation such as the well-known U.S. Genetic Information Nondiscrimination Act of 2008 (Rothstein 2018).

Although the genomic data does not seem to have any single characteristic that makes it categorically different from, say, other biometrics or from family history information, the combination of a number of characteristics makes it difficult to predict the scope of future uses—both socially beneficial and socially harmful—to which this type of data could be put (Naveed et al. 2015). It not only facilitates new insights into disease and risk, but also potentially opens up new types of discrimination, and not only provides new ways to research family connections, but also risks unintended disclosure of sensitive paternity or family relationship information. Naveed and coauthors have proposed a set of six characteristics that together constitute the distinctiveness of genetic information, namely its:

1. relevance to health and behavior,
2. immutability over time,
3. uniqueness among people,
4. mystique in the public consciousness,
5. value in terms of its information content, and
6. kinship information with respect to blood relatives (Naveed et al. 2015).

To determine the degree to which data protection should provide for rules or guidance specific to genomics, it is helpful to examine how these rules apply in specific data protection contexts, such as in determining the identifiability of genomic data. Although a number of general principles around identifiability will sometimes be relevant, such as analyzing the safeguards provided by general-purpose encryption or in determining the possibility that a data subject might be identified by linking the data with other available data sets, a number of other genomic-specific considerations come into play. Some projects, for example, the International Cancer Genome Consortium, have determined that in their contexts, variants in somatic mutations are generally not sufficiently identifiable so as to be considered personal data (ICGC DCC 2015). And the growing scholarly literature on the residual identifiability of the aggregate results of genomic analysis quite clearly provides practical lessons that would be impossible to independently derive from guidance on the notion of personal data in general on its own.

Given the difficulty in interpreting rules in this area, and the proliferation of genetic nondiscrimination safeguards, it seems preferable that safeguards specific to genomic data be considered legitimate, when appropriate, while also ensuring that specialized guidance on compliance with general data protection norms be available for genomic research projects. Given the increasingly international scope of this work the next section turns to the question of transfer in the genomic context.

## Legal compliance and international transfer

Given the historical influence of Europe in the field of data protection in general, and unparalleled international focus on the new GDPR in particular, this section of the article discusses key vehicles through which the GDPR can allow transfer outside the EU to be justified: (A) consent, (B) adequacy, (C) standard contractual clauses, (D) binding corporate rules, and (E) codes of conduct. This article does not discuss the additional obligations, such as those related to record-keeping, that are required when transfer occurs (GDPR Article 30(1)(e), (2)(c)).

### A. Consent

It may be surprising to begin this discussion with consent rather than adequacy but, until now, this approach has been an apparently attractive one for international genomics projects. First, researchers must generally obtain participant consent to biomedical research in any event, so extending that consent to cover international transfer of the participants’ data costs them little. Second, broad consent for research purposes is recognized in European law and reaffirmed by the GDPR (Recital 33). Finally, consent seems to provide uniform coverage that avoids the unevenness of measures such as adequacy decisions, which are unlikely to cover all participating research projects within a consortium as it grows to include projects in countries without a data protection mechanism that has been deemed adequate.

Yet despite these advantages, researchers should be cautious about relying on this transfer justification under the GDPR. The text of the consent exception to transfer requires that “the data subject has explicitly consented to the proposed transfer, after having been informed of the possible risks of such transfers for the data subject due to the absence of an adequacy decision and appropriate safeguards”. This vehicle appears in the GDPR as one of a handful of “[d]erogations for specific situations”. It is, therefore likely to be interpreted narrowly. It is thus not clear to what degree using consent as a basis for transfer is compatible with using broad consent as a lawful basis for processing personal data. Guidance from European data protection authorities under the previous Data Protection Directive discouraged consent as a basis for any “transfers of personal data which might be qualified as repeated, mass or structural” (Article 29 Working Party 2005).

As noted in the contribution of Taylor, Wallace and Prictor to this volume, a tendency is growing toward conceiving of consent as irrelevant—neither necessary nor sufficient—in the data protection analysis when it relates to research, at least in the United Kingdom. The GDPR explicitly envisions the possibility of broad consent, however, in the context of personal data processing for research purposes, although this approach is now subject to stricter scrutiny and may require additional safeguards (Article 29 Working Party 2018, p. 28; Marelli and Testa 2018). Regulatory guidance on this issue notes that the GDPR itself lists data minimization, anonymization, and data security as potential safeguards (Article 29 Working Party 2018, p. 29). It adds transparency as the research progresses as another possible safeguard to offset the absence of specific consent, such as designating a specific person that participants can contact with their questions over time, or providing them with a comprehensive research plan before they consent. The need for alternative safeguards may be greater still when broad consent is paired with international transfer, particularly in the context of attempts to rely on the consent derogation to justify transfer in the context where the processor is also relying on broad consent to personal data processing.

The public interest and legitimate interest justifications for transfer in Article 49(1) are much more circumscribed than they are as justifications for lawful processing in Article 6(1). Article 49(1)(d), for example, specifies that the transfer must be “necessary for important reasons of public interest” (emphasis added).

The recent enforceability of the GDPR provides a convenient excuse for many genomic and health projects to move away from consent as a justification for international sharing of personal data. For the reasons provided above, in most circumstances it is now best avoided.

### B. Adequacy

As noted above in “From the OECD to the GDPR”, adequacy has been the cornerstone of EU data protection justifications for transfer since the arrival of the Data Protection Directive in 1995. Although an adequacy decision is the preferred and usually the most reassuring basis for transfer, adequacy has three primary weaknesses. First, not all countries have been approved (European Commission 2018). Second, even when working in a country with an approved mechanism, the mechanisms that have been approved as adequate in countries like Canada and the United States only cover the entities are subject to those mechanisms. Third, now that the landmark Schrems case invalidated an adequacy decision and now that the GDPR mandates periodic review of such decisions, those who rely on adequacy can no longer simply hope that once approved, their adequacy decision will remain in place indefinitely.

Schrems arose in the wake of Edward Snowden’s revelations about mass state surveillance by United States government agencies and their counterparts within the other countries in the Five Eyes alliance, and the perception of willing collaboration by large technology companies including Google and Facebook. The European Union took the impact on fundamental rights of the programs revealed particularly seriously (European Parliament 2013).

This was the context in which Austrian student Max Schrems launched his legal challenge to the European Commission decision that had previously deemed the U.S./EU Safe Harbour Framework to provide adequate data protection. But indiscriminate surveillance was also explicitly cited by the European Court of Justice in its decision in *Schrems* as a key justification for its invalidation of the prior adequacy decision (European Court of Justice 2015).

In the context of broader discussions of adequacy in the health and genomics context, it has been suggested the EU adequacy approval process is incoherent or even biased (Stoddart et al. 2016). A data protection statute in the Canadian province of Quebec, for example, received negative feedback from EU authorities when it sought adequacy status despite that many feel that it provides stronger protection than PIPEDA, a federal Canadian data protection statute that had already gained adequacy approval. The authors point to a number of such examples.

But there seems nonetheless to be a way to make sense of the apparent inconsistencies in these processes, namely the watershed changes in the public and regulatory understanding of the importance of data protection that accompanied the 2012 Snowden revelations and the privacy scandals that have persisted since, through the EU *Schrems* decision and up to the recent Cambridge Analytica revelations (Osborne and Parkinson 2018). Seen in this broader context, what seems to emanate from the EU regulatory decisions is a clear and understandable tendency toward subjecting adequacy applicants to stricter scrutiny post-2012, especially due to the increasingly meaningful protections provided by EU law. Now that the GDPR mandates periodic review of continued adequacy rather than one-time approval, the developments in the coming years are likely to reveal whether EU decisions owe more to changing standards than to other factors.

When an adequacy decision is available in the circumstances, it remains the preferred mechanism to comply with transfer obligations. Because the decisions generally cover legal frameworks of general application, in these situations virtually no extra effort is required to operationalize adequacy as justification. Because adequacy has recently become a status that can not only be conferred, but also revoked, and with an unclear amount of notice, if any, a contingency plan may be advisable, such as the ability to shift to standard contractual clauses. In the case where the adequacy decision is available but not automatic, for example through self-certification under the EU/U.S. Privacy Shield, or by deliberately bringing a project under the application of Canada’s PIPEDA, which applies only to “commercial activities”, the advantages of adequacy will have to be more carefully weighed against the other transfer justification options available.

### C. Standard contractual clauses

A third way to comply with the GDPR’s transfer rules is by incorporating standard “model clauses” that have been previously approved by the EU Commission, into binding contracts between the sender and recipient (GDPR Article 45(2)(c)–(d)). They were widely cited as the leading alternative transfer mechanism to consider by participants in the U.S./EU Safe Harbour framework after its adequacy approval was revoked in 2016 by the European Court of Justice in the *Schrems* decision (McLelland and Hellmuth 2015; Kugele and Garcia Ward 2015).

It bears noting at this point that the organization whose reliance on Safe Harbour was specifically put into question in Schrems was Facebook. The significance of this is that Facebook used model clauses as the justification for transfers following *Schrems*, which led the plaintiff in the case to initiate a new legal challenge to the decision that had found that those clauses provided adequate protection, along similar lines to what had been argued with respect to Safe Harbour. This challenge, often referred to as Schrems II, is again before the Court of Justice.

In short, in situations where adequacy is an unavailable or impractical basis for transfer, standard contractual clauses will often be the next best option to consider. Practically, this means integrating a set of approved standard clauses into a legally binding agreement between the entities involved in the transfer. Standard clauses are sometimes seen as inflexible. Although it is true that inconvenient portions cannot be omitted or even contradicted or undermined by other clauses, it is possible to supplement the clauses with additional rules that frame the relationship, even clauses that clarify the terms in the standard clauses. The more ambitious the “clarification”, however, the greater the risk of crossing over into a situation where the standard clauses may be undermined or their spirit contradicted, thereby eliminating the desired legal justification for transfer.

### D. Binding corporate rules

Although they are included among the GDPR’s preferred justifications for transfer, BCRs are only rarely likely to be helpful in the context of genomics research. Their intended use is in the context of large supranational organizations or groups of organizations who may need to transfer personal data across borders while staying within the organization’s own internal divisions (Article 47 GDPR). A set of BCRs need to be put into place within the organization that meet the requirements of Article 47 of the GDPR, that must then be externally approved by the relevant data protection authority. On top of requiring these relatively time-consuming, complicated, and expensive initial investments, the approach will often not be the best-suited transfer mechanism given the organizational structure of international genomic research projects, or at least those that are undertaken by partnerships or consortia that include member projects that remain distinct and nationally based.

Unless they are carried out by very large multinational organizations, Binding Corporate Rules are unlikely to be the preferred transfer justification for genomics and health initiatives.

### E. Codes of conduct

The GDPR now also provides that an organization’s adherence to a code of conduct aimed at a specific sector that has been approved by the European Commission according to the processes set out in the GDPR, when paired with binding and enforceable commitments to apply the appropriate safeguards, constitute an independent justification allowing personal data to be transferred to that organization. The central drawback to this approach is that no relevant code of conduct currently exists.

Another limitation of the Code approach is that, although adherence with an approved Code of Conduct provides evidence of compliance with the GDPR generally, it does not provide *proof* of compliance. In other words, it remains theoretically possible to be found to have violated the GDPR even assuming perfect adherence to an approved Code of Conduct. The role of such a Code is not to supplant the GDPR’s obligations, only to clarify and assist in interpreting them in a particular context.

The road to adoption of a European-wide Code of Conduct under the GDPR involves consultation with relevant stakeholders as a text is drafted, the creation and accreditation of an enforcement entity with sufficient independence and capacity and, ultimately, approval by the European Commission.

These hurdles do not appear to be insurmountable. The GDPR provides new incentives to encourage the development of such codes that were not present under the previous *Data Protection Directive*, and BBMRI-ERIC is already leading the coordination of a Code for health (Litton 2017). If the genomics sector and related sectors succeed in putting such a code in place, it has the strong possibility of becoming the most attractive transfer mechanism for data-sharing projects involving many countries. The Code would be available for use by projects anywhere in the world, irrespective of their jurisdiction having sought or received adequacy status. The two benefits of such a Code would be, first, avoiding the persistent legal uncertainty facing the more general transfer mechanisms and, second, beyond international transfer specifically, providing practical certainty in the form of guidance on how general data protection rules in general apply in the specific context of genomics and health.

## Conclusion

The first half century of the field of data protection developed in such a way that the key framework, when it comes to the international transfer of data, are the rules set out in Chapter V of the GDPR.

Among the options available to genomics and health research initiatives looking to comply with these rules, adequacy decisions should be the first option to consider, when existing decisions cover all of the relevant jurisdictions in question. Alternatively, initiatives are likely to have to make do with standard contractual clauses. In the near future, however, stakeholders in translational genomics and health would best be served by pooling its expertise and resources to create its own code of conduct, in which it can help shape the interpretations of the GDPR’s rules, keep them up to date as interpretations progress, and ensure that the expectations are clear.

From a broader perspective, this article noted the underlying policy goal of achieving the condition necessary to allow the elimination of all regulation of international data sharing: namely the guarantee that adequate data protection will be assured wherever the data may end up. Although this overall goal remains distant, we can move toward it by working toward achieving it in the genomics and health sector specifically. Although it remains an ambitious aim, an international Code of Conduct approved under the GDPR would bring us near the finish line.

In the meantime, careful attention is still needed to the country-specific implementations and interpretations of key aspects of data protection. The following contributions in this volume not only give us insight into this variation, but they also assist us in envisioning the contours of the substantive content that could be included in a genomic data protection Code of Conduct that reaches beyond Europe, and has global aspirations.
